# Inhibition of glycogen synthase kinase-3 beta induces apoptosis and mitotic catastrophe by disrupting centrosome regulation in cancer cells

**DOI:** 10.1038/srep13249

**Published:** 2015-08-21

**Authors:** Yuki Yoshino, Chikashi Ishioka

**Affiliations:** 1 Department of Clinical Oncology, Institute of Development, Aging and Cancer, Tohoku University, Seiryo-machi 4-1, Aoba-ku, Sendai 980-8575, Japan; 2 Department of Medical Oncology, Tohoku University Hospital, Tohoku University, Seiryo-machi 1-1, Aoba-ku, Sendai 980-8574, Japan

## Abstract

Glycogen synthase kinase-3 beta (GSK-3β) has been investigated as a therapeutic target for numerous human diseases including cancer because of their diverse cellular functions. Although GSK-3β inhibitors have been investigated as anticancer reagents, precise biological mechanisms remain to be determined. In this study, we investigated the anticancer effects of GSK-3β inhibitors on cancer cell lines and observed centrosome dysregulation which resulted in abnormal mitosis. Mitotic checkpoints sensed the mitotic abnormalities and induced apoptosis. For cells that were inherently resistant to apoptosis, cell death distinct from apoptosis was induced. After GSK-3β inhibitor treatment, these cells exhibited characteristic features of mitotic catastrophe, including distended and multivesiculated nuclei and inappropriate reductions in cyclin B1 expression. This suggested that mitotic catastrophe was an alternative mechanism in cells resistant to apoptosis. Although the role of GSK-3β in centrosomes has not yet been clarified, phosphorylated GSK-3β was localised in centrosomes. From these data, GSK-3β seems to regulate centrosome function. Thus, we propose that centrosome dysregulation is an important mechanism for the anticancer effects of GSK-3β inhibitors and that mitotic catastrophe serves as a safe-guard system to remove cells with any mitotic abnormalities induced by GSK-3β inhibition.

Glycogen synthase kinase-3 beta (GSK-3β) was first identified as a negative regulator of glycogenesis and was subsequently found to regulate various signalling pathways and cellular functions^1^. For example, as a key regulator in the Wnt/β-catenin pathway, GSK-3β phosphorylates β-catenin to induce the degradation of β-catenin in cooperation with adenomatous polyposis coli gene product^2^. GSK-3β also phosphorylates various proteins involved in regulating the cell cycle, apoptosis, and survival, such as cyclin D1, MYC, BAX, and NF-κB^3^,^4^. Furthermore, SNAI1, an important transcription factor involved in the epithelial-mesenchymal transition, was found to be a substrate of GSK-3β^5^. In general, GSK-3β phosphorylates its substrates, inducing the degradation of the substrates or inhibition of their enzymatic activities.

Due to its wide range of functions, GSK-3β is believed to be involved in various disease processes, including neurodegenerative diseases, diabetes mellitus, and cancer. Although GSK-3β affects the signalling pathways that regulate the proliferation and survival of cancer cells, the precise role of GSK-3β in cancer pathophysiology remains controversial. Because some GSK-3β substrates are key proteins for promoting cell proliferation and survival, such as β-catenin and cyclin D1^6^, GSK-3β is considered as a tumour suppressor. However, a recent report showed that higher GSK-3β expression was related to a worse prognosis in those with non-small cell lung cancer^7^. In tumorigenesis, GSK-3β has important roles in development and cancer cell maintenance in leukaemia^8^ and glioblastoma^9^. In addition, several reports showed that GSK-3β inhibitors induced misaligned chromosomes on the metaphase plate and mitotic spindle deformation^10^,^11^,^12^,^13^. Misaligned chromosomes due to GSK-3β inhibition was, in part, mediated by γ-tubulin complex proteins (GCPs)^11^ or CRMP1^13^. GSK-3β might regulate chromosome constitution to prevent chromosomal instability. These data suggest that GSK-3β has tumour promoting activity in some situations. Based on these results, GSK-3β may change its role at different stages of carcinogenesis. Otherwise, GSK-3β may be bivalent in nature.

Because of its relevance to various disease processes, GSK-3β is considered to be an attractive target for drug development for several diseases, including neurodegenerative diseases like Alzheimer’s disease, diabetes mellitus, and cancer^2^,^3^,^14^,^15^. Regarding neurodegenerative diseases, inhibiting GSK-3β results in reduced phosphorylation of several proteins, such as tau, which subsequently protects neurons^15^,^16^,^17^. Because GSK-3β regulates the activities of glycogen synthase and other enzymes involved in regulating glucose metabolism, GSK-3β inhibitors are anticipated to ameliorate diabetes^3^.

For cancer treatment, GSK-3β inhibition has been studied as a possible therapeutic strategy. GSK-3β knockdown or using GSK-3β inhibitors has been shown to inhibit cancer cell proliferation in pancreatic^18^,^19^, prostate^20^, and colon^21^ cancers, and leukaemia^22^. Additionally, contributions by the NF-κB pathway^23^,^24^,^25^,^26^ and the mitochondrial apoptosis pathway^27^,^28^ were reported to be involved in the antiproliferative effects of GSK-3β inhibition in cancer cells. However, the exact mechanism involved is controversial and remains to be elucidated.

In this study, we investigated the molecular and biological responses to a GSK-3β inhibitor by various cancer cell lines to identify the primary molecular pathway responsible for its antiproliferative effects.

## Results

### Effects of AR-A014418 on cancer cell proliferation and survival

**Figure 1:**
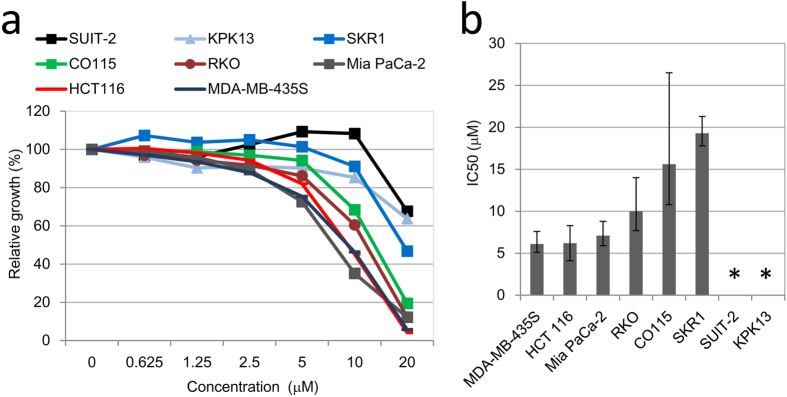
AR-A014418 antiproliferative effects. (**a**) Representative data for AR-A014418 growth inhibitory effects on eight cancer cell lines. Cell counts were determined after cells were exposed to AR-A014418 at indicated concentrations for 120 h. Cell growth relative to the control (DMSO) was determined. (**b**) IC50 values of cell lines at 120 h after adding AR-A014418. IC50 values were determined from at least three independent experiments using a logistic regression model. Error bars indicate 95% confidence intervals (CIs) (*>30 μM).

To investigate the inhibitory effects of a GSK-3β inhibitor on cancer cell proliferation, cell proliferation was determined after long-term (120 h) treatment with AR-A014418, a specific GSK-3β inhibitor^17^ (Fig. 1a). IC50 values were determined using a logistic regression analysis from at least three independent experiments (Fig. 1b). Based on their IC50 values, we selected five cell lines for following study: HCT 116, MDA-MB-435S; and RKO as sensitive cell lines, and KPK13 and SUIT-2 as relatively insensitive cell lines. Shorter treatment (72 h) with AR-A0114418 did not show significant growth suppression below 20 μM (data not shown).

**Figure 2:**
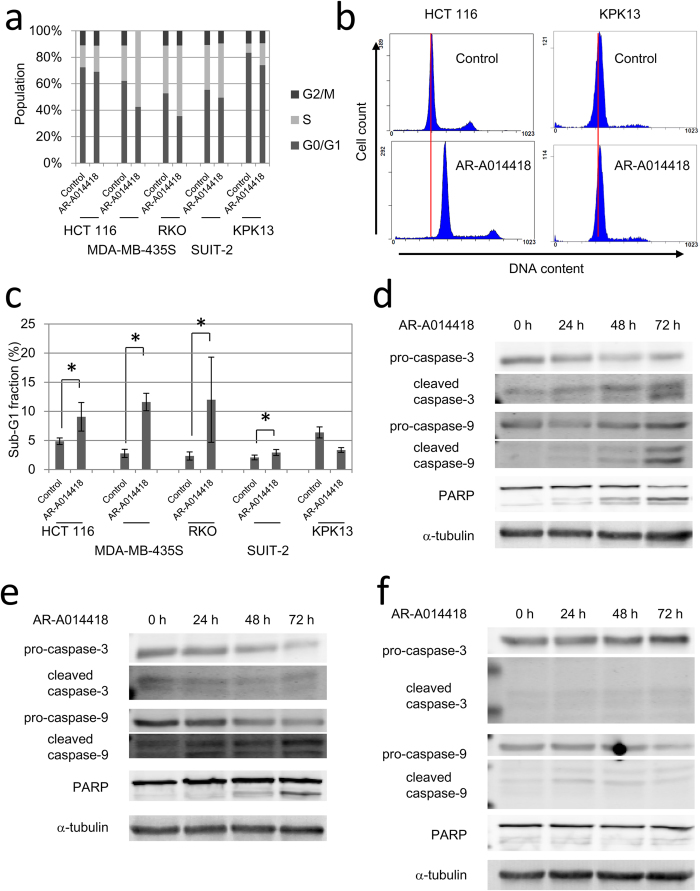
Cell cycle changes and apoptosis induction after AR-A014418. (**a**) Changes in cell cycle distribution after AR-A014418 treatment. Cells were analysed by flow cytometry after treatment with 20 μM AR-A014418 for 120 h. Average of three independent experiments is shown. (**b**) Representative DNA histograms of HCT 116 and KPK13 after 120 h treatment with 20 μM AR-A0114418. Red lines indicate peaks of G0/G1 population in control samples. (**c**) Sub-G1 fractions of cells lines after AR-A0114418 treatment. Cells were analysed after treatment with 20 μM AR-A0114418 for 120 h. Sub-G1 fractions increased for all cell lines except for KPK13 cells. Error bars indicate 95% CIs (*p < 0.05). (**d,e,f**) Western blot of apoptosis related proteins in RKO (**d**), HCT116 (**e**), and MDA-MB-435S (**f**). Cells were treated with 20 μM AR-A0114418 for indicated periods.

To clarify what type of cell death occurred, we examined cell cycle distributions and apoptosis-related protein expression after AR-A0114418 treatment. Cell cycle distributions were analysed after long-term (120 h) treatment with AR-A014418 at 20 μM, which was sufficiently higher than IC50s of three sensitive cell lines. AR-A014418 treatment significantly increased the S phase fraction and correspondingly reduced the G0/G1 phase fraction for all cell lines, which indicated cell cycle arrest at promotion to M phase (Fig. 2a). Figure 2b shows representative DNA histograms of sensitive and insensitive cell lines, HCT 116 and KPK13, respectively. AR-A014418 treatment also increased the sub-G1 fraction for all cell lines, except for KPK13 cells (Fig. 2c).

We next investigated for activation of apoptosis pathways at several time points after adding 20 μM AR-A0114418. AR-A014418 treatment resulted in increased levels of cleaved caspase-3, cleaved caspase-9, and PARP cleavage fragments in RKO cells over time (Fig. 2d). These results indicated that apoptosis pathways had been activated. In HCT 116 cells, similar results were observed after AR-A014418 treatment (Fig. 2e). The short turnover time of cleaved caspase-3 in this cell line appeared to be a reason for why an increase in cleaved caspase-3 was not detected despite the apparent decrease in pro-caspase-3 in HCT 116. In contrast, caspase activation and PARP cleavage were not detected in MDA-MB-4335S cells that were treated with AR-A014418, despite their high sensitivity to AR-A014418 (Fig. 2f). In the two insensitive cell lines, little or no cleaved caspase-3, cleaved caspase-9, or PARP cleaved fragments were detected (Supplementary Fig. S1).

### Four established signalling pathways are not downregulated after AR-A014418 treatment

To determine which signalling pathway was responsible for the antiproliferative effects of AR-A014418 treatment, we investigated the Wnt/β-catenin and the NF-κB signalling pathways, which are reported to be regulated by GSK-3β.

**Figure 3:**
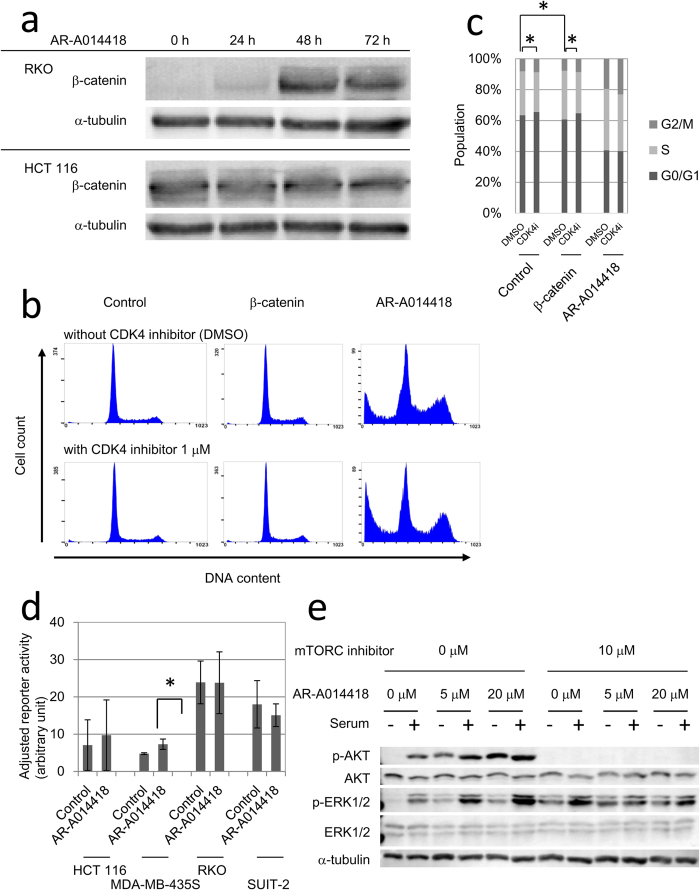
AR-A014418 effects on the Wnt/β-catenin, NF-κB, MAPK, and PI3K/AKT signalling pathways. (**a**) β-catenin accumulation by AR-A0114418 in RKO and HCT 116. Cells were harvested for immunoblotting after treatment with 20 μM AR-A014418 for indicated periods. (**b**) Comparisons of DNA histograms between β-catenin-overexpressing and AR-A014418-treated HeLa cells. A CDK4 inhibitor (1 μM) was added at 24 h after transfection with a mutant (S33Y) β-catenin expression vector or after adding 20 μM AR-A014418. After another 48 h, cells were harvested for cell cycle analysis. (**c**) Cell cycle distributions of HeLa cells subjected to mutant β–catenin overexpression or AR-A014418 treatment with or without a CDK4 inhibitor. Cells were treated in the same way as (**b**) Average of three independent experiments is shown. Error bars indicate 95% CIs (*p < 0.05). (**d**) NF-κB reporter activities with or without AR-A014418 treatment. Cells were pre-treated with DMSO or 20 μM AR-A0114418 for 48 h from the next day of reporter plasmid transfection. After the treatment, cells were allowed to release MMPs into fresh medium for 24 h. (*p < 0.01) (**e**) AKT and ERK phosphorylation after AR-A014418 treatment. Cells were cultured in serum-free media with AR-A014418 at the indicated concentrations for 48 h. After serum starvation, cells were stimulated with 10% serum for 30 min and then harvested for western blot.

To investigate the possible involvement of the Wnt/β-catenin signalling pathway, we assessed changes in β-catenin expression induced by AR-A014418 treatment. β-catenin levels began to increase at 48 h after adding 20 μM AR-A014418 to RKO cells with wild-type β-catenin^29^ but not in HCT 116 cells with mutant β-catenin which has deletion of serine 45 residue, target of GSK-3β^29^ (Fig. 3a). However, accelerated rather than suppressed cell proliferation was observed after forced expression of wild-type or constitutively active mutant β-catenin (S33Y) in RKO and HCT 116 (Supplementary Fig. S2a). To clarify the difference of overexpression and AR-A0114418 induced upregulation of β-catenin, we utilized HeLa, which harbours no mutation in Wnt/β-catenin pathway. The DNA histogram of cells with forced β-catenin expression was distinct from that of cells treated with 20 μM AR-A0114418 (Fig. 3b). Administering a cdk4 inhibitor in conjunction with β-catenin overexpression reversed these changes in cell cycle distribution, although the cdk4 inhibitor could not counteract the effects of AR-A014418 (Fig. 3c).

Next, tο investigate whether the NF-κB pathway was responsible for the antiproliferative effects of AR-A014418, we measured cellular NF-κB activities using a dual-luciferase reporter assay. The normalized baseline NF-κB activity did not appear to reflect the sensitivity to AR-A014418 in all cell lines except for KPK13 (Fig. 3d). Furthermore, pre-treatment with 20 μM AR-A014418 for 48 h did not significantly reduce normalized NF-κB activities in any of these cell lines (Fig. 3d). Transfection of reporter plasmids was too toxic and too inefficient for KPK13 to evaluate NF-κB reporter activity (data not shown). These results suggested that Wnt/β-catenin and the NF-κB signalling pathway were not downstream effectors of AR-A014418.

We also investigated the effects of AR-A014418 treatment on the mitogen activated protein kinase (MAPK) pathway and the PI3K/AKT which are important for cell proliferation and survival. We examined the phosphorylation status of ERK1/2 and AKT in HeLa, which harbours no mutation in both pathways. Both phospho-ERK1/2 and phospho-AKT levels were increased after 20 μM AR-A014418 treatment in HeLa cells (Fig. 3e). Phospho-AKT upregulation after AR-A014418 treatment was independent of serum stimulation, whereas that of phospho-ERK1/2 was dependent on this stimulation. ERK and AKT phosphorylation levels were also upregulated in HCT 116 and RKO cells, although the degree of phosphorylation was less than that in HeLa cells (Supplementary Fig. S2b). AKT phosphorylation completely disappeared after treatment with an mTORC inhibitor, whereas that of ERK was not affected (Fig. 3e). These data suggested that GSK-3β inhibition had increased the AKT phosphorylation level by disrupting the AKT/GSK-3β/mTORC2 feedback signalling loop. We thought that these changes in MAPK and PI3K/AKT pathways by AR-A0114418 were not sufficient to explain the antiproliferative effects of AR-A014418 because activation of these pathways usually promotes cell growth and survival.

### AR-A0114418 induces mitotic spindle dysfunction and chromosomal instability

**Figure 4:**
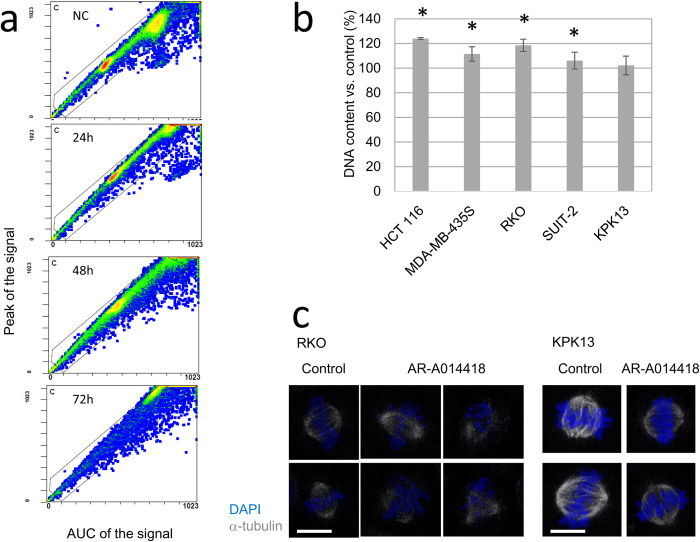
Chromosomal instability induced by AR-A0114418. (**a**) Representative plots of cellular DNA contents in RKO cells analysed by flow cytometry. Cells were analysed at the indicated times after adding 20 μM AR-A014418. Signal peaks indicate maximum DNA densities in each cell. Areas under the curve (AUC) of the signal indicate the total DNA contents in each cell. The ratio of the signal peak to AUC of the signal is an indicator of particle aggregation. When cells make aggregates, the AUC of the signal increases whereas the signal peak does not. (**b**) Increases in DNA contents after treatment with AR-A014418. Peak signal intensity of G0/G1 phase in DNA histogram was measured as a DNA content after 120 h treatment with 20 μM AR-A0114418. Average of three independent experiments is shown. Error bars indicate 95% CIs (*p < 0.05). (**c**) Mitotic spindle morphology after AR-A014418. Cells were treated with 20 μM AR-A014418 for 48 h and then stained. (Scale bar = 10 μm).

To determine the mechanism underlying the antiproliferative effects of the GSK-3β inhibitor, the DNA histograms were analysed further. In addition to the changes in cell cycle distributions, cell fraction peaks were shifted towards higher DNA contents after 72 h treatment with 20 μM AR-A0114418 (Fig. 4a). Except for KPK13 cells, there were significant increases in the cellular DNA contents in all cell lines after 120 h treatment with AR-A0114418 (Fig. 4b). FISH analysis using probes specific for chromosomes 17 and 20 showed random amplification of these chromosomes after treatment with 20 μM AR-A0114418, which suggested chromosomal instability (Supplementary Fig. S3).

To investigate the mechanism underlying these chromosomal instabilities, mitotic spindles were visualized by immunocytofluorescent staining for α-tubulin. Abnormal spindles, such as mono- and tri-pole spindles, and spindles without poles, appeared in all cell lines except for KPK13 cells after 48 h treatment with 20 μM AR-A014418 (Fig. 4c, Supplementary Fig. S4). In KPK13 cells, the most insensitive cell line, AR-A014418 did not induce any spindle abnormalities (Fig. 4c, Supplementary Fig. S4). Similar spindle abnormalities were observed after treatment with GSK-3β inhibitor XXVI^30^ and lithium chloride, which are GSK-3β inhibitors structurally distinct from AR-A014418 (Supplementary Fig. S5a). Taken together, abnormal mitosis with deformed mitotic spindles appeared to be the cause of the chromosomal instability induced by GSK-3β inhibition.

### GSK-3β inhibition disrupts centrosome regulation

**Figure 5:**
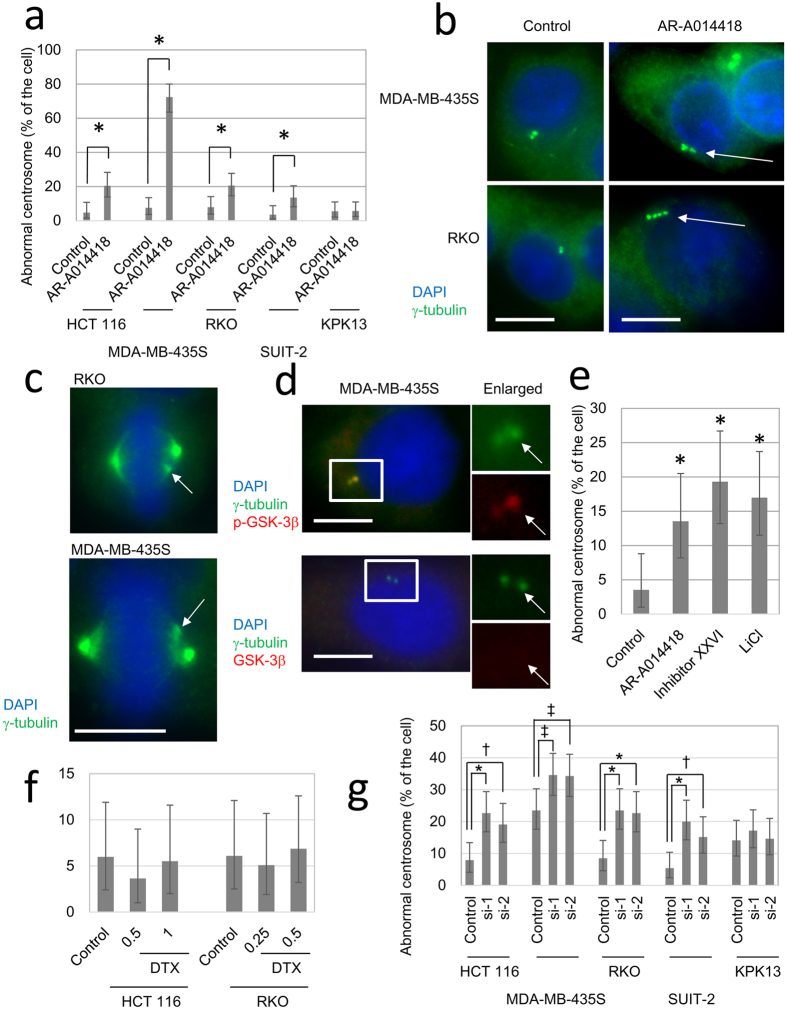
Centrosome dysregulation by GSK-3β inhibitors. (**a**) Frequency of abnormal centrosomes. Cells were treated with 20 μM AR-A014418 for 48 h and then stained with an anti-γ-tubulin antibody. At least 100 cells were examined in each sample. CIs and p-values were calculated by Fisher’s exact test. Error bars indicate 95% CIs. (*p < 0.01) (**b**) Representative examples of centrosome abnormalities in interphase cells. Arrows indicate aberrant centrosomes. (Scale bar = 10 μm) (**c**) Representative examples of centrosome abnormalities in mitotic **c**ells. Arrows indicate extra-pole. (Scale bar = 10 μm) (**d**) Centrosome localization of phospho-GSK-3β. MDA-MB-435S cells were stained with an anti-γ-tubulin antibody and an anti-GSK-3β or anti-phospho-GSK-3β antibody. The right column shows higher power images of the boxed areas in the left column images. Arrows indicate centrosomes. (Scale bar = 10 μm) (**e**) Frequency of centrosome abnormalities induced by three distinct GSK-3β inhibitors. Cells were examined after 48 h treatment with AR-A014418 (20 μM), GSK-3β inhibitor XXVI (Inhibitor XXVI, 10 μM), or lithium chloride (LiCl, 50 mM). The data was obtained and analysed by the same way as (**a**). Error bars indicate 95% CIs (*p < 0.01). (**f**) Frequency of centrosome abnormalities after docetaxel treatment. Cells were treated with docetaxel at their IC50s (0.5 nM for HCT 116 and 0.25 nM for RKO) or higher concentration for 48 h. The data was obtained and analysed by the same way as (**a**). (**g**) Frequency of centrosome abnormalities after GSK-3β knock-down. Centrosomes were counted 72 h after transfection of siRNA. The data was obtained and analysed by the same way as (**a**). (*p < 0.001, †p < 0.005, ‡p = 0.02).

Centrosomes participate in the formation of mitotic spindles^31^. To investigate any changes in centrosomes induced by AR-A014418, we used immunocytofluorescent staining for γ-tubulin, one of the major components of centrosomes. Treatment with 20 μM AR-A0114418 for 48 h significantly increased the numbers of cells with more than two centrosomes or with morphologically abnormal centrosomes in all cell lines, except for KPK13 cells (Fig. 5a). Representative images of these centrosome abnormalities are shown in Fig. 5b. In addition, pseudo-dipole formation, which one of the sequelae of centrosome amplification^32^, was found in RKO and MDA-MB-435S cells treated with AR-A0114418 (Fig. 5c).

GSK-3β reportedly localizes at centrosomes and spindle poles^10^. To confirm the localization of GSK-3β in the cell lines, we observed localization of GSK-3β, γ-tubulin and α-tubulin by immunocytostaining. Phosphorylated GSK-3β co-localised with γ-tubulin-positive centrosomes in MDA-MB-435S cells (Fig. 5d). In contrast, an anti-GSK-3β antibody that cannot determine phosphorylation status was relatively diffusely distributed and did not co-localize with γ-tubulin (Fig. 5d). Similar localization patterns were observed in HCT 116 and RKO cells (data not shown). These data confirmed the localization of GSK-3β in centrosomes and suggested that GSK-3β possibly worked on centrosomes to regulate mitotic spindle formation.

To preclude that centrosome aberration induced by AR-A0114418 was a non-specific effect of the agent, we examined centrosomes in cells treated with GSK-3β inhibitor XXVI and lithium chloride. These GSK-3β inhibitors also induced centrosome abnormalities similar to those induced by AR-A014418 treatment in MDA-MB-435S cells (Fig. 5e). These GSK-3β inhibitors also induced centrosome abnormalities in HCT 116 and RKO (Supplementary Fig. S5b). In contrast, docetaxel, direct microtubule toxin, did not induce centrosome abnormality at IC50 or higher concentration in RKO and HCT 116 (Fig. 5f) although it induced mitotic spindle deformation (data not shown).

To further confirm the role of GSK-3β in centrosome regulation, we observed centrosome changes after knock-down of GSK-3β using RNAi. Two siRNAs, but not control siRNA, successfully knocked down GSK-3β in all five cell lines examined (Supplementary Fig. S6a). In the cell lines except for KPK13, rates of cells with abnormal centrosomes were significantly increased after GSK-3β knock-down (Fig. 5g, Supplementary Fig. S6b). These data strongly supported our hypothesis that inhibiting GSK-3β activity was responsible for inducing the centrosome aberrations and the subsequent mitotic spindle deformations.

### Mitotic abnormalities induce apoptotic and non-apoptotic cell death

**Figure 6:**
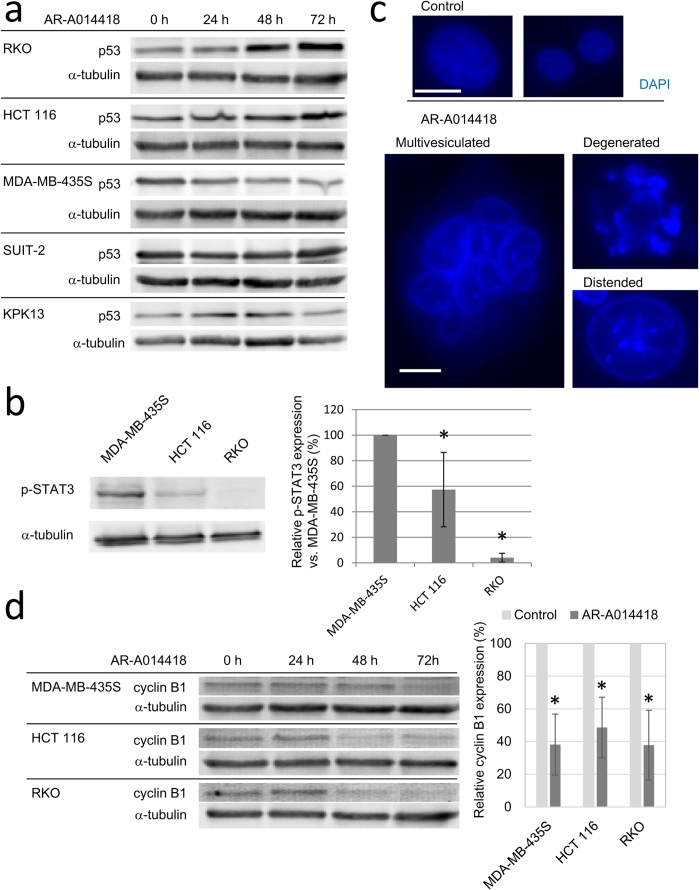
The way of cell death induced by AR-A0114418. (**a**) Protein expression of p53 after AR-A014418 treatment. Cells were harvested for western blot after treatment with 20 μM AR-A014418 for indicated periods. (**b**) Phospho-STAT3 expression in sensitive cell lines. Band intensities were determined by densitometry and the expression level relative to that in MDA-MB-435S cells was determined. Average of three independent experiments is shown in the right graph. Error bars indicate 95% CIs. (**c**) Morphology of nuclei in MDA-MB-435S after AR-A0114418 treatment. Cells were fixed and stained with DAPI after 48 h treatment with 20 μM AR-A014418. (Scale bar = 10 μm) (**d**) Protein expression of cyclin B1. Cells were harvested for western blot after treatment with 20 μM AR-A014418 for indicated periods. Right graph shows relative cyclin B1 expression compared with control after 72 h treatment. Average of three independent experiments is shown. Error bars indicate 95% CIs. (*p < 0.001).

The biochemical markers of apoptosis, including cleavage of caspases and PARP, were observed in HCT 116 and RKO cells but not in MDA-MB-435S cells. As a cause of this difference, we first focused on p53 status. HCT 116 and RKO cells have wild-type p53, whereas MDA-MB-435S cells have mutant p53^33^,^34^,^35^. In fact, treatment with 20 μM AR-A014418 increased the p53 protein levels by 48 h after adding of AR-A0114418 in HCT 116 and RKO cells but not in MDA-MB-435S cells (Fig. 6a). Insensitive cell lines, SUIT-2 and KPK13 did not increase p53 expression after AR-A0114418 treatment (Fig. 6a). In addition, phosphorylated STAT3, which negatively regulates apoptosis, was expressed at significantly higher levels in MDA-MB-435S cells than in HCT 116 and RKO cells (Fig. 6b). Based on these data, it was suggested that MDA-MB-435S was resistant to apoptosis that should be induced by centrosome aberration after GSK-3β inhibition.

Because apoptosis was not responsible for MDA-MB-435S cell death, we investigated if mitotic catastrophe, another mechanism of cell death caused by severe spindle dysfunction^36^,^37^,^38^, was responsible for cell death in MDA-MB-435S. At the morphological level, mitotic catastrophe is characterized by distorted, multivesiculated, or distended nuclei and at the molecular level by inappropriate cyclin B1 degradation, even with spindle abnormalities^36^,^37^. In MDA-MB-435S cells, relatively large, dilated nuclei that were highly distorted or multivesiculated appeared after 48 h treatment with 20 μM AR-A014418 (Fig. 6c). In contrast, apoptotic bodies were not found in MDA-MB-435S cells after treatment with AR-A014418. Similar morphological abnormalities were observed in cells treated with GSK-3β inhibitor XXVI and lithium chloride (data not shown). Although AR-A014418 treatment induced similar changes in HCT 116 and RKO cells, typical apoptotic bodies were also observed in these cells (Supplementary Fig. S7). We also examined cyclin B1 expression as a marker of mitotic catastrophe. Cyclin B1 expression was decreased by 48 h after adding 20 μM AR-A014418. After 72 h treatment, cyclin B1 expression was significantly lower in MDA-MB-435S, HCT 116, and RKO cells that had been treated with 20 μM AR-A014418 compared to the levels in control cells (Fig. 6d). GSK-3β inhibitor XXVI and lithium chloride also decreased cyclin B1 expression in MDA-MB-435S cells after 72 h treatment (Supplementary Fig. S8).

### GSK-3β inhibition do not develop chromosomal instability in normal cells

**Figure 7:**
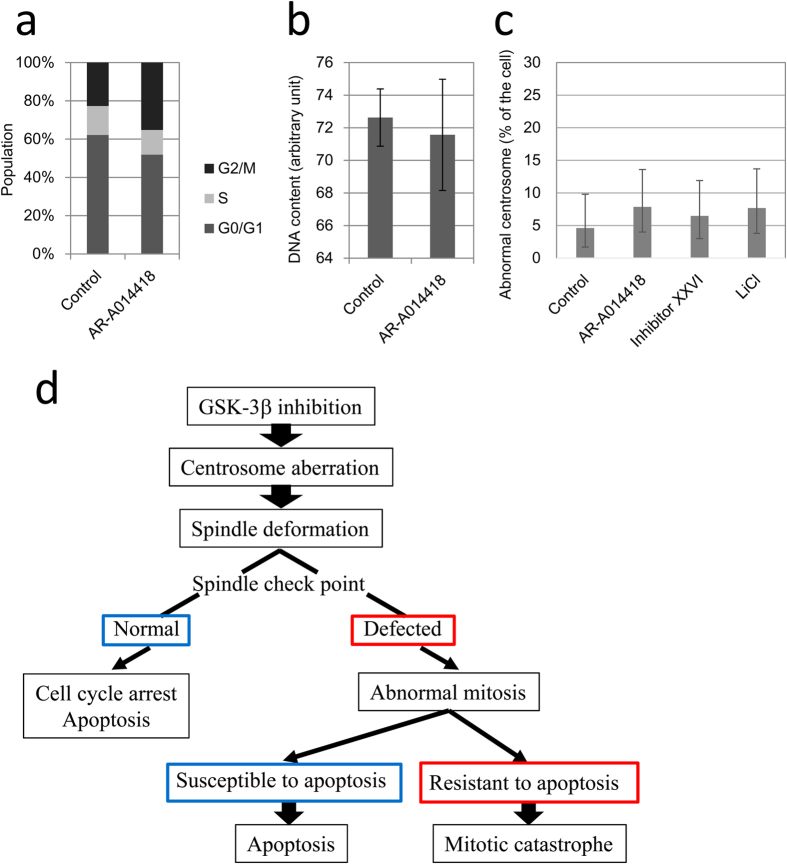
Effects of AR-A014418 on normal cells. (**a**) Cell cycle distributions of HUVEC cells. Cells were analysed after treatment with DMSO or 20 μM AR-A014418 for 96 h. Average of three independent experiments is shown. (**b**) Changes in cellular DNA contents after treatment with 20 μM AR-A014418 for 96 h. Error bars indicate 95% CIs. (**c**) Frequency of abnormal centrosomes. Cells were stained after treatment with AR-A014418 (20 μM), GSK-3β inhibitor XXVI (10 μM), or lithium chloride (50 mM) for 42 h. At least 100 cells were examined in each sample. CIs and p-values were calculated by Fisher’s exact test. Error bars indicate 95% CIs. (**d**) Schematic overview of cellular responses to GSK-3β inhibition.

To investigate if GSK-3β inhibitors induced chromosomal instability in normal cells, we examined cell cycle distributions, cellular DNA contents, and centrosomes in HUVEC cells after treatment with the GSK-3β inhibitors. Cell cycle analysis showed an apparent M-phase arrest in HUVEC cells treated with 20 μM AR-A014418 for 96 h (Fig. 7a). Because longer-term treatment (120 h) resulted in overt cell death, cell cycle distribution was not evaluable (data not shown). An analysis of the cell cycle histograms did not show increase in cellular DNA contents (Fig. 7b). Furthermore, there was no increase in the frequency of cells with abnormal centrosomes after treatment with any of the three GSK-3β inhibitors, AR-A014418, GSK-3β inhibitor XXVI, and lithium chloride (Fig. 7c). Based on these data, GSK-3β inhibitors apparently did not induce any chromosomal instability in HUVEC cells.

## Discussion

In this study, we found that inhibition of GSK-3β induced two modes of cell death, apoptosis and mitotic catastrophe, subsequent to centrosome aberrations (Fig. 7d). GSK-3β inhibitors disrupted centrosome regulation and caused deformations in mitotic spindles. This resulted in chromosome mis-segregation and, ultimately, chromosomal instability. These mitotic abnormalities induced apoptotic cell death in those cell lines with an intact apoptosis pathway. In addition, these abnormalities also induced mitotic catastrophe in cell lines with deficient apoptosis pathways.

To investigate a mechanism for the antiproliferative effects of a GSK-3β inhibitor, we examined the activation status of the Wnt/β-catenin, NF-κB, MAPK and, PI3K/AKT signalling pathways, which are known to be important for cell proliferation and survival. Although, none of these pathways was consistently inhibited by AR-A0114418 at the concentration higher than IC50s. In contrast, the MAPK and PI3K/AKT pathways were activated by the inhibitor. Although this was evident in HeLa, upregulation was less obvious in HCT 116 and RKO. This seemed to be because HCT 116 and RKO cells have a G13D mutation in *KRAS* and a V600E mutation in *BRAF* respectively^39^, which over-activates the MAPK pathway. In HCT 116 and RKO cells, over-activation of the MAPK pathway could have suppressed AKT phosphorylation by feedback regulation. Based on these data, we considered that these four signalling pathways were not responsible for the antiproliferative effects of AR-A0114418.

Although the previously mentioned signalling pathways failed to explain the effects of AR-A0114418, we identified centrosome aberration as a possible mechanism of antiproliferative effects. GSK-3β inhibition induced centrosome aberration and subsequently resulted in abnormal mitosis in the most cell lines. This was confirmed by three structurally distinct GSK-3β inhibitors and knock-down of GSK-3β. In contrast, docetaxel, which directly inhibits microtubule depolymerisation, destroyed mitotic spindles without any centrosome abnormalities. These data suggested that the centrosome aberration was the cause, not a result of the mitotic abnormalities. A number of reports showed similar results that GSK-3β inhibitors induced misaligned chromosomes on the metaphase plate and mitotic spindle deformation^10^,^11^,^12^,^13^. Misaligned chromosomes due to GSK-3β inhibition was, in part, mediated by CRMP1, one of the microtubule associating proteins^13^. Phosphorylated GSK-3β localizes at centrosomes during interphase and spindle poles during mitosis^10^. In the cell lines we used, GSK-3β was also localised at centrosomes. Knocking down a centrosome component, such as γ-tubulin complex proteins (GCPs), results in abnormal spindle formation and chromosome misalignments^11^. GCP5, one of these GCPs, was reportedly associated with GSK-3β. GCP5 is one of the components of γ-tubulin ring complex (γ-TuRC), which is the main constituent of peri-centriolar matrix (PCM). Recent studies revealed PCM and other centrosome components such as Aurora A and ninein participate in regulation of centriole replication^40^,^41^. Because some of aforementioned centrosome proteins, such as GCP5 have a consensus motif of GSK-3β, GSK-3β may phosphorylate these proteins to regulate centrosomes. Based on our data and the results in previous reports, GSK-3β possibly works on centrosomes to control centrosome functions, including the formation of mitotic spindles. Due to the differences from docetaxel treatment and the localization of GSK-3β, a GSK-3β inhibitor would not be a direct microtubule poison, but would act as a “centrosome poison”, possible new class of anticancer reagents.

As a result of centrosome dysregulation after GSK-3β inhibition, we observed mitotic abnormality and chromosomal instability. We observed mitotic spindle deformations and misaligned chromosomes at mitotic metaphase in cells treated with GSK-3β inhibitors. Flow cytometry and FISH analyses showed induction of increases in cellular DNA contents and random chromosome amplifications, suggestive of chromosomal instability. These chromosomal instability seemed to be the result of mitotic abnormalities.

Cells which underwent abnormal mitosis died of two different modes. Typical apoptosis characterized by the activation of caspases, PARP cleavage, and the appearance of apoptotic bodies was observed for HCT 116 and RKO cells treated with AR-A0114418. In contrast, no apoptosis markers were seen in MDA-MB-435S cells. Instead, distended and multivesiculated nuclei were found in MDA-MB-435S cells. In addition, cyclin B1 expression was decreased by the three distinct GSK-3β inhibitors, even though mitotic spindles were deformed. Because these changes are typically seen in mitotic catastrophe, one mode of cell death that is distinct from apoptosis^36^,^37^,^38^, mitotic catastrophe was thought to be a primary mode of cell death in MDA-MB-435S after GSK-3β inhibition. Although the features of mitotic catastrophe were also found in HCT 116 and RKO cells, apoptosis appeared to be the major mode of their cell death.

Previous reports indicated that STAT3 over-activation increased surviving^42^ and induced resistance to apoptosis by MDA-MB-435S cells^43^. In addition, MDA-MB-435S lacks functional p53. An inherent resistance to apoptosis might make MDA-MB-435S cells susceptible to death due to mitotic catastrophe compared to HCT 116 and RKO. In other words, mitotic catastrophe may be a safe-guard mechanism to remove cells that escape apoptosis without correcting abnormalities in their mitotic machinery.

Chromosomal instability significantly contributes to carcinogenesis^44^. Because AR-A0114418 induces DNA aneuploidy and chromosomal instability in cancer cells, there was concern regarding possible carcinogenic activity by GSK-3β inhibition in healthy tissues. We used HUVEC cells as normal cells to assess this possibility. AR-A0114418 caused M-phase arrest but did not increase the cellular DNA contents in HUVEC cells. In addition, all three distinct GSK-3β inhibitors did not increase the frequency of centrosome aberration. This may have been because healthy mitotic checkpoints effectively remove cells that are undergoing abnormal mitosis before centrosome amplification and any increase in cellular DNA contents becomes observable. We currently cannot verify this hypothesis because we do not know the earliest events that take place after GSK-3β inhibitor treatment or the direct molecular target of GSK-3β in centrosomes. When the direct target is found, we will be able to investigate the effects of GSK-3β inhibitors on healthy cells.

In this study, we had not identified a direct molecular target of GSK-3β. Based on our data and the results in previous reports, the target appears to be in centrosomes and GSK-3β acts as a regulator of centrosome maintenance, spindle formation, and microtubule dynamics. It also needs to be determined why AR-A0114418 induced centrosome amplification at a much higher frequency in MDA-MB-435S cells than in other cell lines and why AR-A0114418 induced virtually no changes in KPK13 cells. The answers to these questions will provide new insights into the machinery of mitosis and cell proliferation. In future studies, the direct molecular target of GSK-3β and the role of GSK-3β in centrosome regulation need to be investigated.

In conclusion, we propose a new molecular mechanism of anti-proliferative effects of GSK-3β inhibitors: GSK-3β inhibition induce centrosome dysregulation and mitotic abnormality, which result in cell death including mitotic catastrophe.

## Methods

### Cell lines and culture

Mia PaCa-2 cells were from the Riken BioResource Centre (Ibaraki, Japan). KPK13^45^, SKR1^46^, and SUIT-2 cells were from the Cell Resource Centre for Biomedical Research (Institute of Aging, Development and Cancer, Tohoku University, Miyagi, Japan). HCT 116, MDA-MB-435S, and RKO cells were from the ATCC (Manassas, VA, USA). CO115 and HeLa cells were kind gifts of Dr. John M. Mariadason (Ludwig Institute for Cancer Research, Melbourne, Australia) and Professor A. Horii (Department of Molecular Pathology, School of Medicine, Tohoku University, Miyagi, Japan), respectively. A human umbilical vein endothelial cell (HUVEC) line was from Takara Bio (Shiga, Japan). HeLa, KPK13, Mia PaCa-2, RKO, SKR1, and SUIT-2 cells were maintained in DMEM supplemented with 8% FBS (Biowest, Nuaille, France). CO115, HCT 116, and MDA-MB-435S cells were maintained in RPMI1640 supplemented with 8% FBS. HUVECs were maintained with an EGM-2 BulletKit with the provided supplements (BD, Franklin Lakes, NJ, USA).

### Chemicals

AR-A014418^17^, the GSK-3β inhibitor XXVI, and a cdk4 inhibitor were from Merck (Darmstadt, Germany). Docetaxel and 5-fluorouracil were from Tokyo Chemical Industries (Tokyo, Japan). KU0063794, LY294002, lithium chloride, and other chemicals not otherwise specified were from Wako Pure Chemical Industries (Tokyo, Japan).

### Cell proliferation assay

Cells were first seeded in 48-well microtiter plates (1 × 10^3^ cells/well), and inhibitors were added 24 h later. After an additional 120 h, medium was replaced with fresh medium supplemented with 10% AlamarBlue (Life Technologies, Carlsbad, CA, USA). After incubation for 1 h, the concentration of the reduced indicator was measured with a FluoroSkan Ascent FL (Thermo, Waltham, MA, USA) with excitation at 544 nm and emission at 590 nm.

### Cell cycle analysis

Cells were seeded at 5 × 10^4^ cells/well in 6-well plates, after which they were incubated with inhibitors for 96 h. After trypsinization, four volumes of ice-cold 100% ethanol were added to a single cell suspension and stored at 4 °C overnight. Fixed cells were centrifuged at 500 g for 5 min and the supernatant was discarded. Cell pellets were re-suspended in PBS that contained 0.1% Triton X-100, propidium iodide (50 μg/ml), and RNaseA (5 μg/ml), and then incubated at room temperature for 30 min. A Cytomics FC500 flow cytometer (Beckman-Coulter, Brea, CA, USA) was used to analyse these samples.

### Western blotting

Cells were seeded in 10-cm dishes (2−8 × 10^4^ cells/dish) and incubated for 24 h, after which inhibitors were added and cells were allowed to grow for the indicated periods of time. Cells were lysed in lysis buffer (25 mM HEPES, pH 7.6, 150 mM NaCl, 1% NP-40, 2% SDS, and 10% sucrose) supplemented with a protease inhibitor cocktail (Roche, Basel, Switzerland) and a phosphatase inhibitor cocktail (5 mM NaF, 200 μM sodium orthovanadate, 1 mM sodium molybdate, 2 mM sodium pyrophosphate, and 2 mM disodium beta-glycerophosphate). Protein concentrations were determined using OPA reagent (Thermo), according to the manufacturer’s instructions. Protein samples were separated by SDS-PAGE using gels prepared with WIDE RANGE Gel Preparation Buffer (Nacalai tesque, Kyoto, Japan). After electroblotting and blocking with skim milk, membranes were first reacted with a primary and then with appropriate secondary antibodies conjugated to fluorescent dyes. An Odyssey Infrared Imaging System (LI-COR Biosciences, Lincoln, NE, USA) was used for signal detection. The primary and secondary antibodies used are listed in Supplementary Table S1. ImageJ v1.49 (http://imagej.nih.gov/ij/) was used for image analysis.

### Reporter assay

For an NF-κB reporter assay, a Ready-To-Glow NF-κB Secreted Luciferase Reporter System (Takara Bio) was used. Cells were seeded at 5 × 10^4^ cells/well at 24 h before transfection. An NF-κB reporter plasmid or a control plasmid driven by the CMV promoter was used for transfection. At 24 h after transfection, medium was replaced with fresh medium that contained inhibitors, after which the cells were incubated for an additional 24 h. Medium was replaced with fresh medium and cells were allowed to release reporters for 24 h. Reporter concentrations in supernatants were determined using an Lmax II 384 luminometer (Molecular Device, Sunnyvale, CA, USA), according to the manufacturer’s instructions.

### Plasmids

Mammalian expression plasmids pcDNA3-β-catenin (ID: 16828) and pcDNA3-S33Y β-catenin (ID: 19286) were from Addgene (Cambridge, MA, USA). Plasmids were amplified in an *Escherichia coli* DH5α strain and purified using a Plasmid Maxi Plus Kit (Qiagen, Limburg, The Netherlands). Plasmids were transfected into cells by Polyethylenimine MAX (Polysciences, Inc., Warrington, PA, USA) according to a manufacturer’s instruction.

### FISH

CEP DNA Probes, FISH probes specific for chromosomes 17 and 20, were from Abbott (Abbott Park, IL, USA). Samples were prepared according to the manufacturer’s instructions, and then observed under the BIOREVOBZ-9000 microscope (Keyence, Osaka, Japan).

### Immunocytofluorescence

For α-tubulin, γ-tubulin, total GSK-3β, and phospho-GSK-3β staining, samples were prepared as described in previous reports^47^,^48^ with minor modifications. The primary and secondary antibodies used are listed in Supplementary Table S1. Mitotic spindles were observed under a Leica TCS SP8 confocal microscope (Leica Microsystems, Wetzlar, Germany). GSK-3β localization and the morphologies of centrosomes and nuclei were observed under a BIOREVOBZ-9000 microscope.

### RNAi

siRNAs were purchased from Life Technologies. Following siRNA were used; Silencer Select Negative Control #1 siRNA, GSK3B Silencer Select Validated siRNA (ID: s6239), and GSK3B Silencer Select Validated siRNA (ID: s6241). siRNA was transfected by Lipofectamine RNAiMAX (Life Technologies) at final concentration of 10 nM according to manufacturer’s instructions.

### Statistical analysis

JMP 11 software (SAS Institute Inc., Tokyo, Japan) was used for statistical analysis. Graphs were constructed using Excel 2013 (Microsoft Corporation, Redmond, WA, USA). Statistical comparisons between two different samples were made by two-tailed Welch’s test. Comparisons between more than two samples were made by ANOVA. When a result of ANOVA indicated significant difference, post-hoc comparisons were made by Dunnett’s test to calculate p-values. To assess the frequency of cells with centrosome abnormalities, at least 100 cells were observed for each sample and the percentages of cells with more than two centrosomes were determined. Statistical comparisons of these percentages between two samples were made by Fisher’s exact test. A p-value of <0.05 was considered significant.

## Additional Information

**How to cite this article**: Yoshino, Y. and Ishioka, C. Inhibition of glycogen synthase kinase-3 beta induces apoptosis and mitotic catastrophe by disrupting centrosome regulation in cancer cells. *Sci. Rep.* **5**, 13249; doi: 10.1038/srep13249 (2015).

## Supplementary Material

Supplementary Information
