# Supplementary material for: Inhibition of glycogen synthase kinase-3 beta induces apoptosis and mitotic catastrophe by disrupting centrosome regulation in cancer cells
Source: Sci Rep. 2015 Aug 21;5:13249. doi: 10.1038/srep13249 (PMC4543981; doi:10.1038/srep13249)
Supplement: Supplementary Information [file srep13249-s1.pdf]

Title: **Inhibition of glycogen synthase kinase-3 beta induces apoptosis and mitotic catastrophe by disrupting centrosome regulation in cancer cells**

Authors: Yuki Yoshino[1], Chikashi Ishioka[1, 2, *]

[1] Department of Clinical Oncology, Institute of Development, Aging and Cancer, Tohoku University, Seiryo-machi 4-1, Aoba-ku, Sendai 980-8575, Japan
[2] Department of Medical Oncology, Tohoku University Hospital, Tohoku University, Seiryo-machi 1-1, Aoba-ku, Sendai 980-8574, Japan

* Correspondence author
Department of Clinical Oncology, Institute of Development, Aging and Cancer, Tohoku University, Seiryo-machi 4-1, Aoba-ku, Sendai, 980-8575, Japan
Tel: +81 227178547; Fax: +81 227178548
Email: chikashi@idac.tohoku.ac.jp

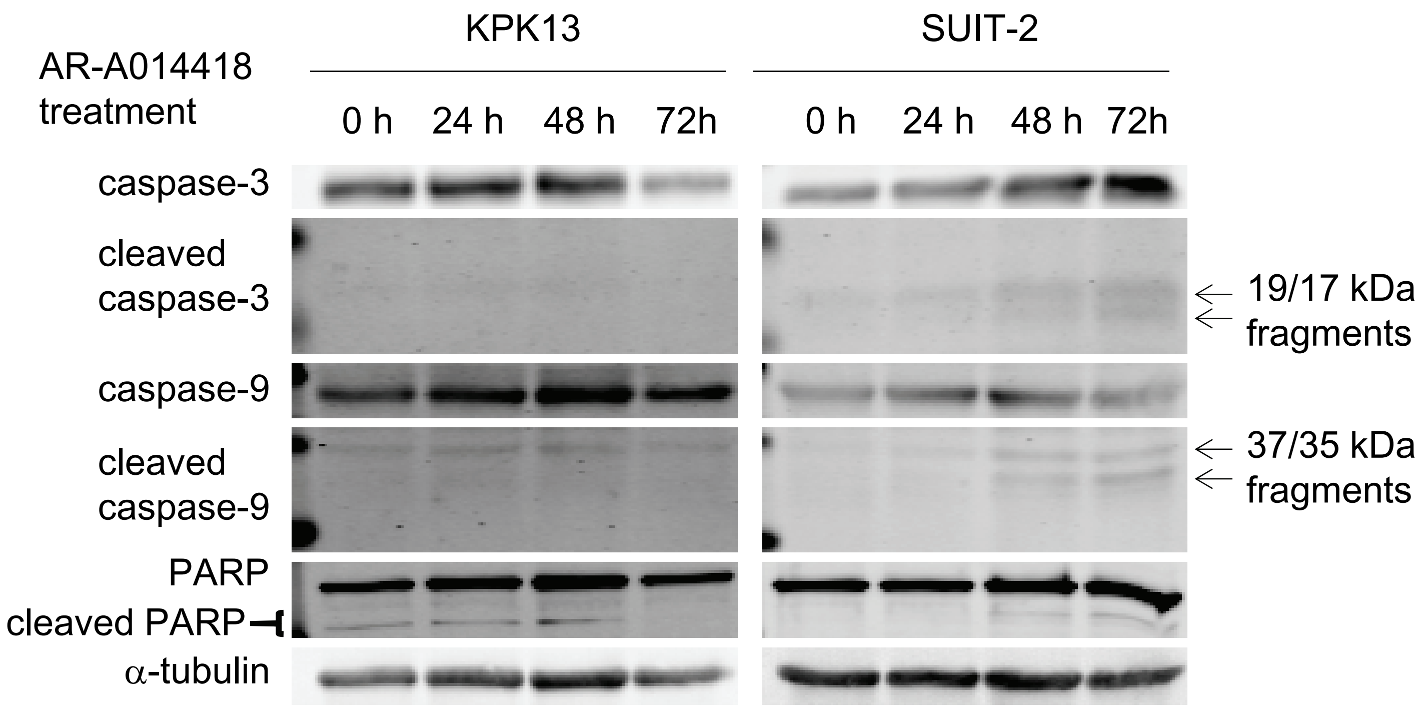

Supplementary Figure S1. Expression of apoptosis related proteins after AR-A0114418 treatment in resistant cell lines. Cells were harvested at the indicated times after adding 20 μM AR-A014418 and subjected to western blot.

a

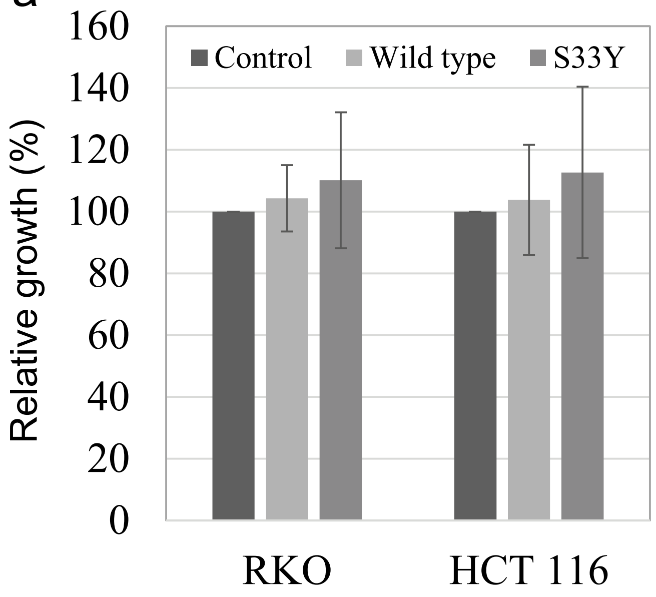

b

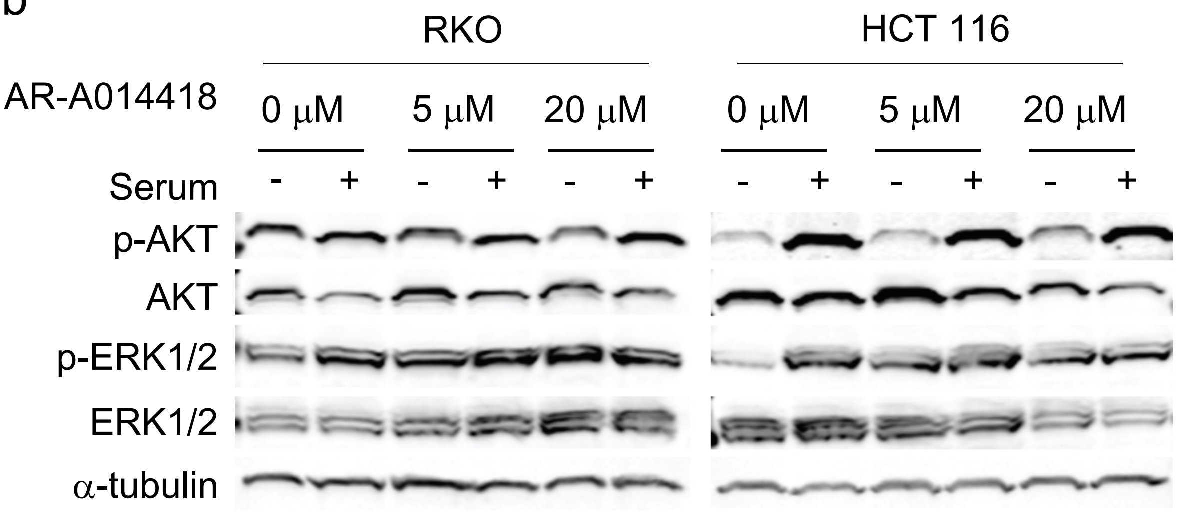

Supplementary Figure S2. Effects of AR-A0114418 on Wnt/β-catenin, PI3K/AKT, and MAPK pathways.
(a) Empty, wild-type β-catenin, or S33Y mutant β-catenin expression vectors were transfected to HeLa cells. Cell cycle was analyzed 96 h after transfection. (b) Cells were treated with AR-A0114418 at indicated concentrations for 48 h.

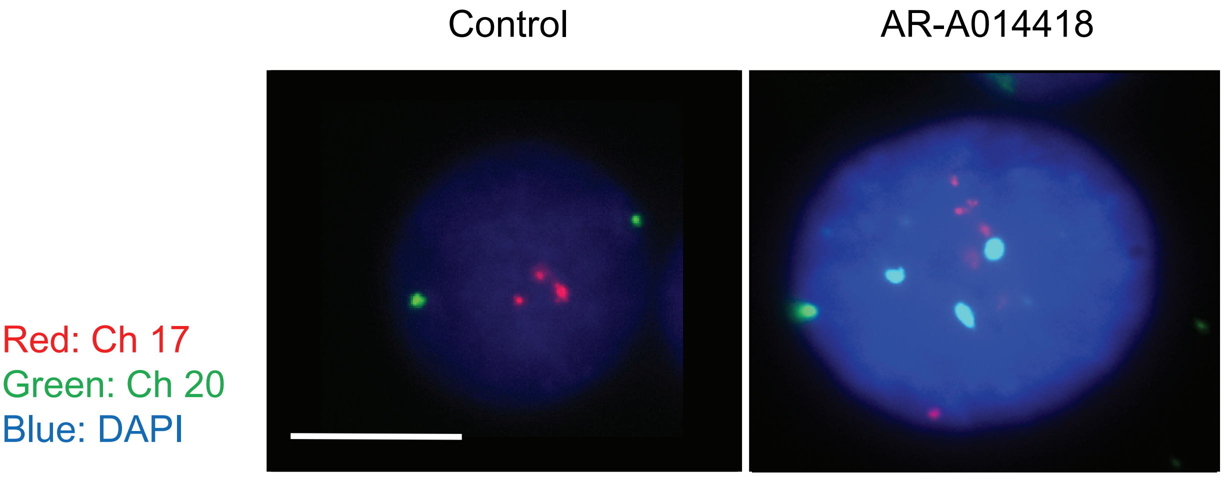

Supplementary Figure S3. FISH analysis after AR-A0114418 treatment.

RKO was treated with DMSO or 20 μM AR-A0114418 for 48 h and stained with probes specific to chromosome 17 and 20. Representative images are shown. Pattern of change in chromosomal number was varied from cell to cell. Because long-term (120 h) treatment disrupted cell morphology, evaluation was difficult. (Scale bar = 10 μm)

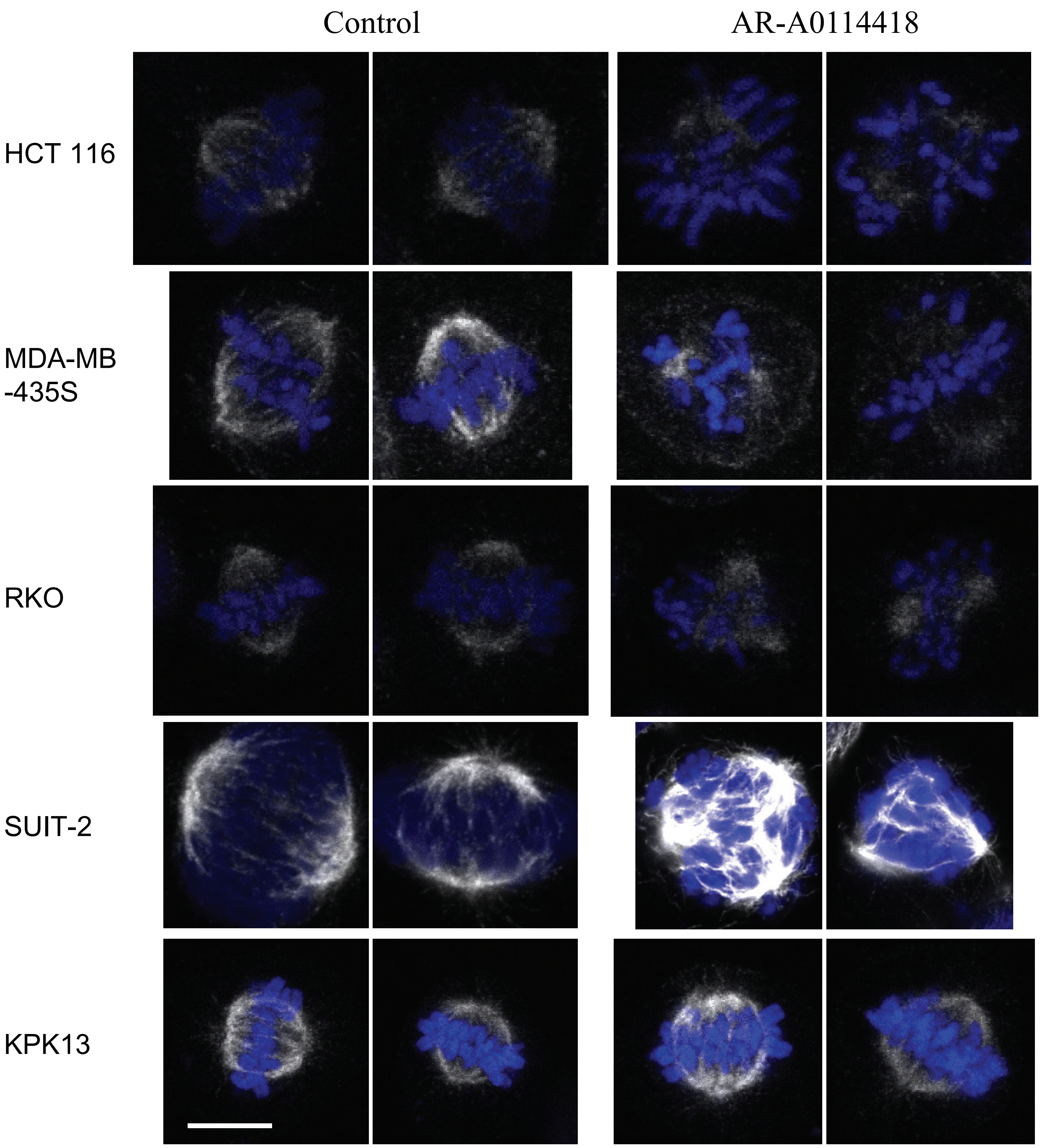

Supplementary Figure S4. Morphology of mitosis after AR-A0114418 treatment.
Each cell was treated with DMSO or 20 μM AR-A0114418 for 48 h. Representative images are shown. (Scale bar = 10 μm)

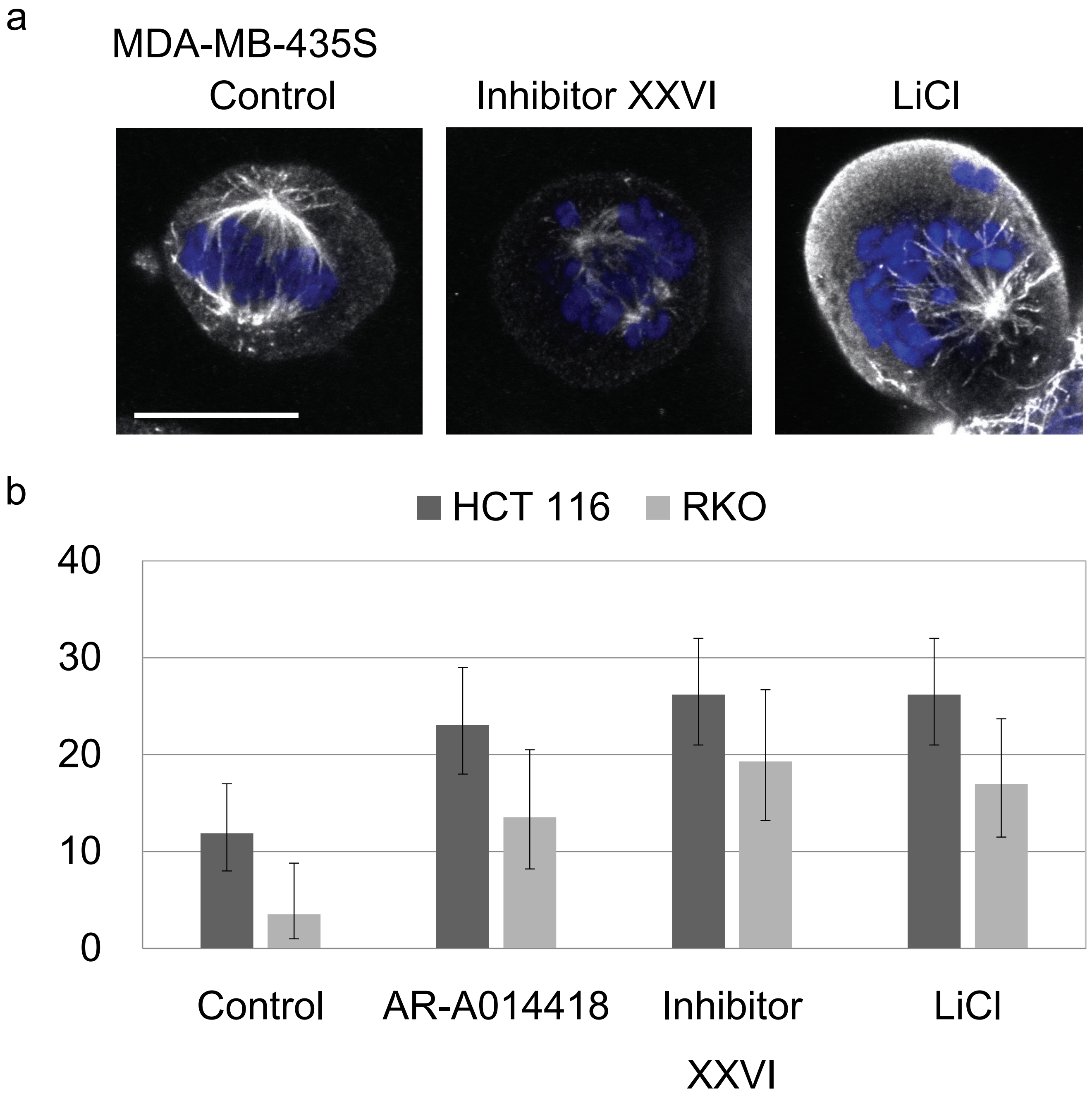

Supplementary Figure S5. Effects of GSK-3β inhibitors distinct from AR-A0114418.
(a) Mitotic spindles after treatment with GSK-3β inhibitors. MDA-MB-435S cells were treated with GSK-3β inhibitor XXVI (Inhibitor XXVI) or lithium chloride (LiCl) for 48 h.
(b) Frequencies of centrosome abnormalities induced by treatment with GSK-3β inhibitors. HCT 116 and RKO Cells were treated with 20 μM AR-A0114418, 10 μM GSK-3β inhibitor XXVI, and 50 mM lithium chloride for 48 h. At least 100 cells were examined in each sample. CIs and p-values were calculated by Fisher's exact test. Error bars indicate 95% CIs (* $p < 0.01$).

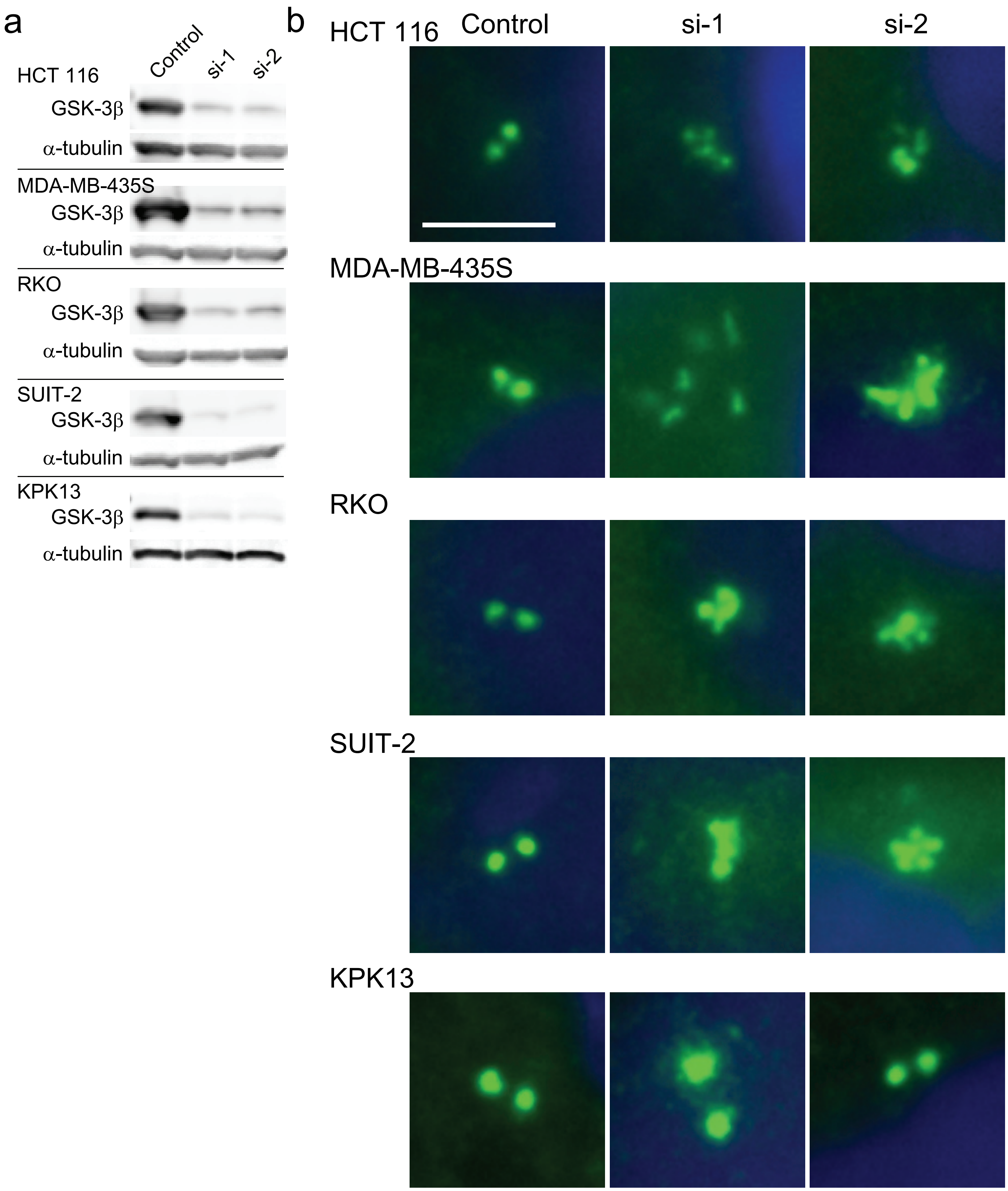

Supplementary Figure S6. Centrosome changes after GSK-3β knock-down.
(a) After 72 h from transfection with 10 nM control or GSK-3β-targeting siRNA, cells were lysed and subjected to western blotting.
(b) Cells were subjected to immunocytostaining 72 h after transfection with 10 nM control or GSK-3β-targeting siRNA. Representative images are shown. (Scale bar = 5 μm)

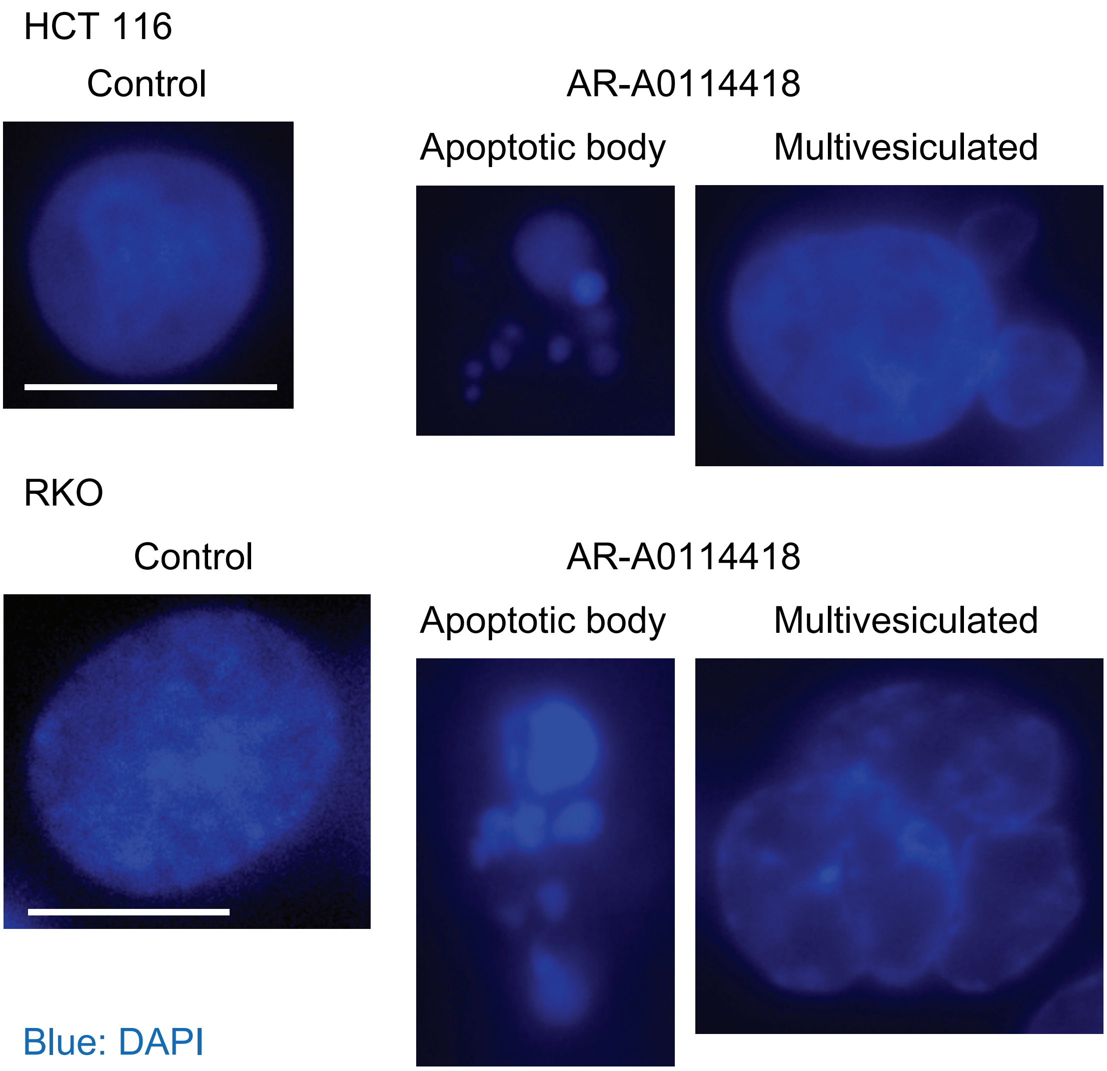

Supplementary Figure S7. Morphology of nuclei after AR-A0114418 treatment.
Each cell was treated with DMSO or 20 μM AR-A0114418 for 48 h. Representative images are shown. (Scale bar = 10 μm)

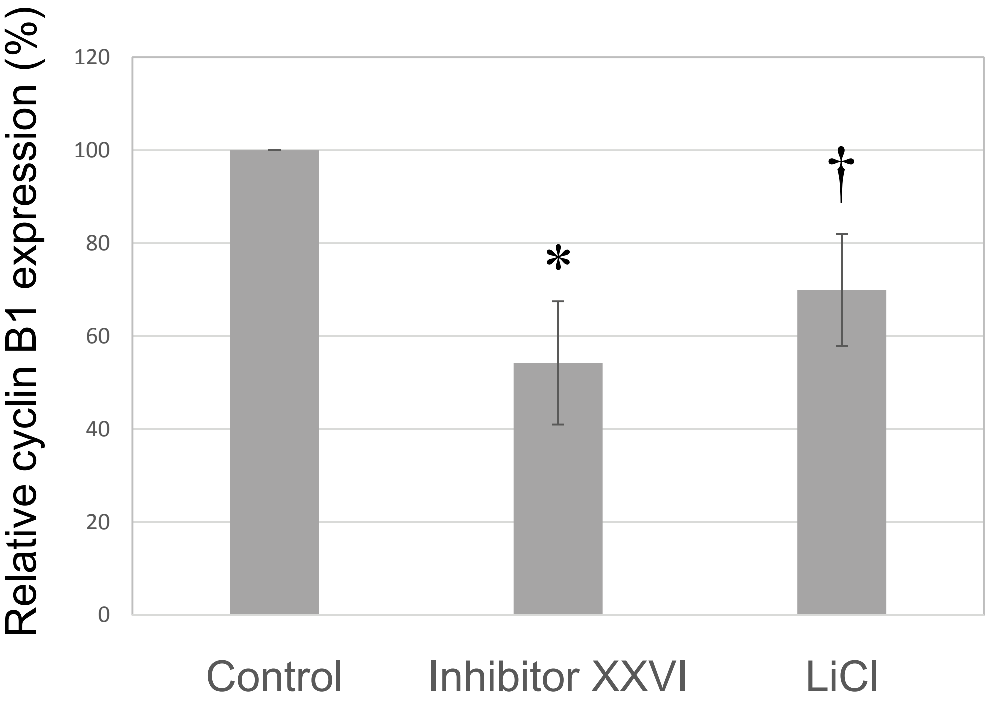

Supplementary Figure S8. Relative protein expression of cyclin B1 after GSK-3β inhibitors.
MDA-MB-435S cells were treated with 10 μM GSK-3β inhibitor XXVI or 50 mM LiCl for 72 h. Error bars indicate 95% CI. (* $p = 0.003$, † $p = 0.024$, compared with control, Dunnett's test)

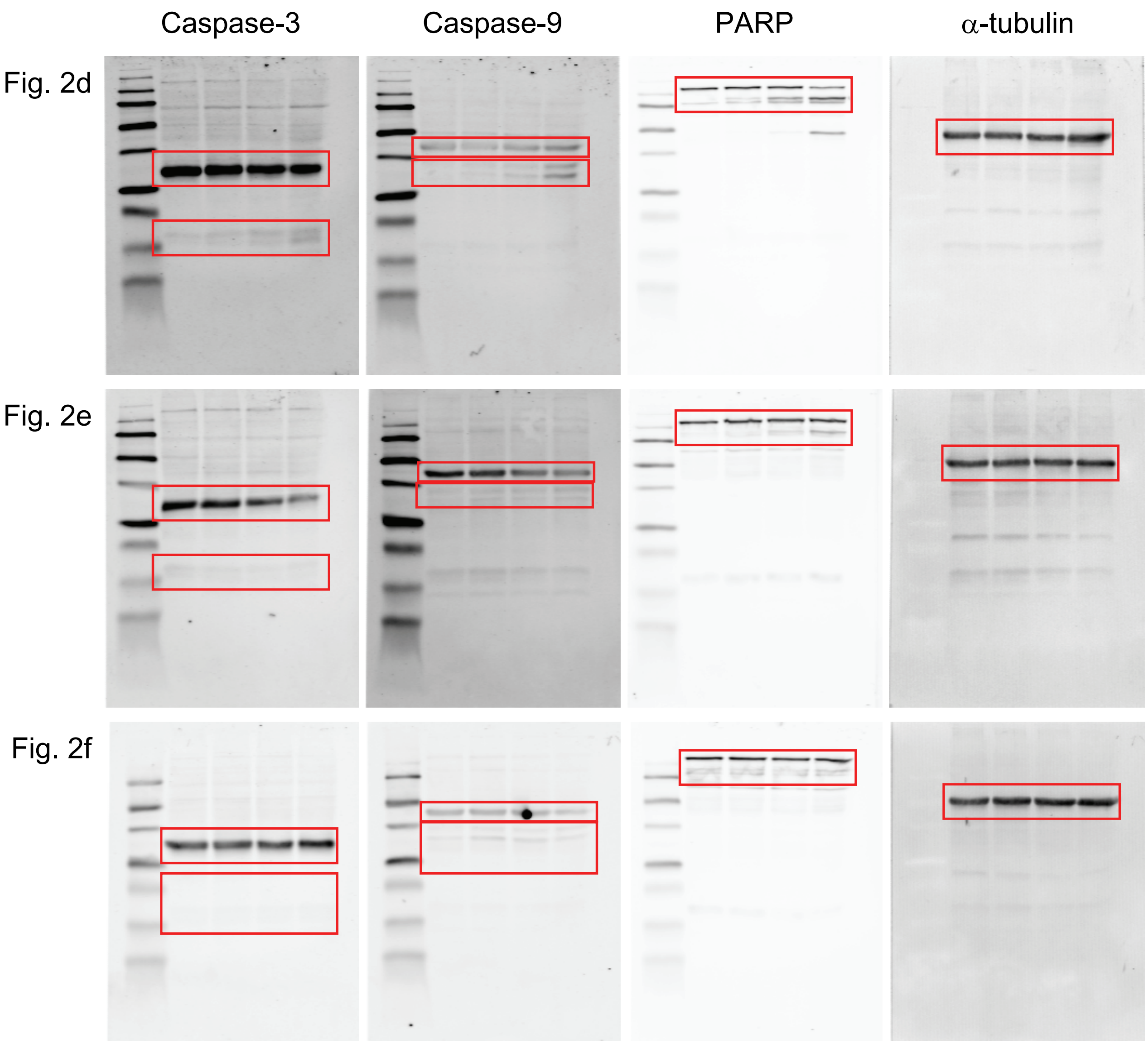

Supplementary Figure S9. Uncropped images of Western blotting of Fig. 2d, e, and f

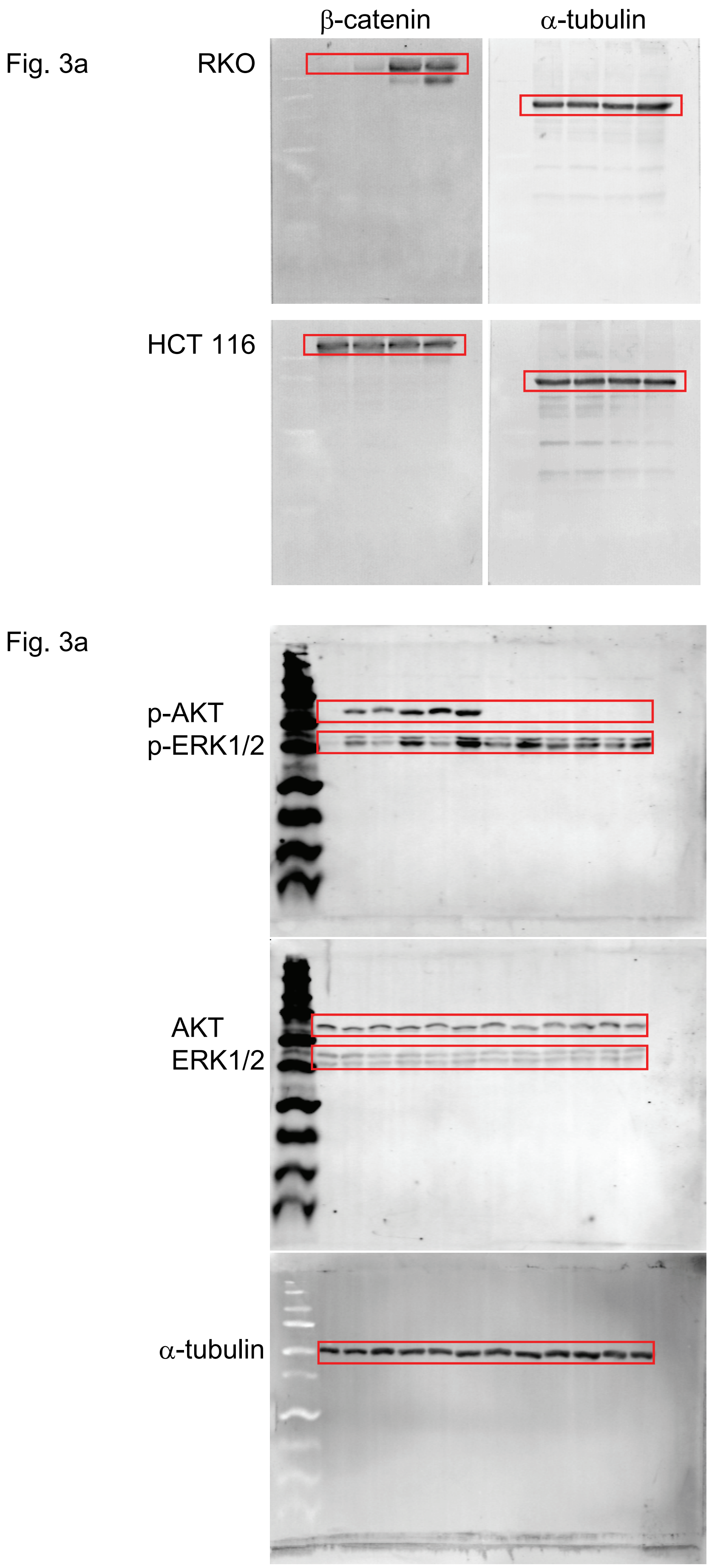

Supplementary Figure S10. Uncropped images of Western blotting of Fig. 3a and e

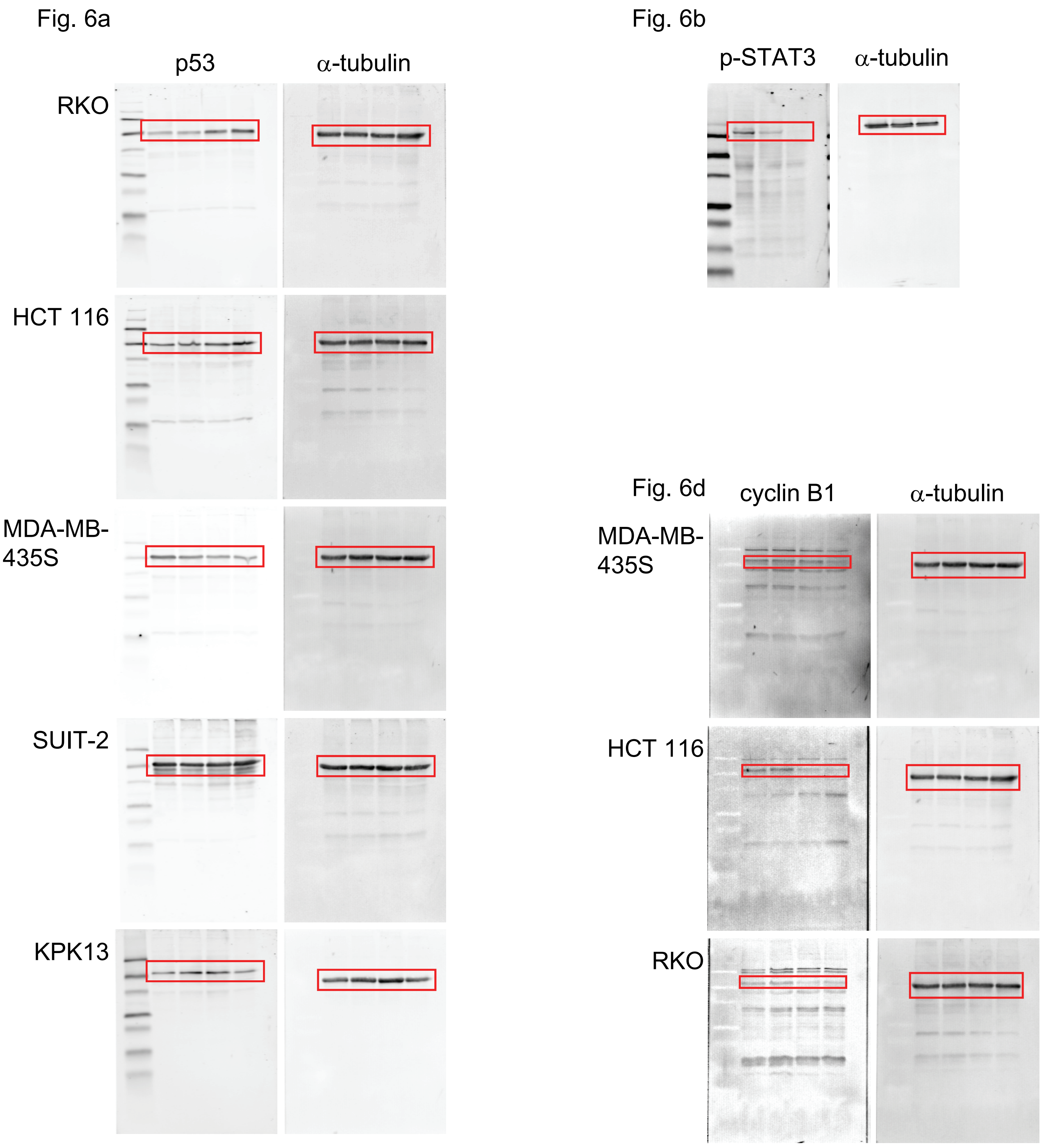

Supplementary Figure S11. Uncropped images of Western blotting of Fig. 6a, b, and d

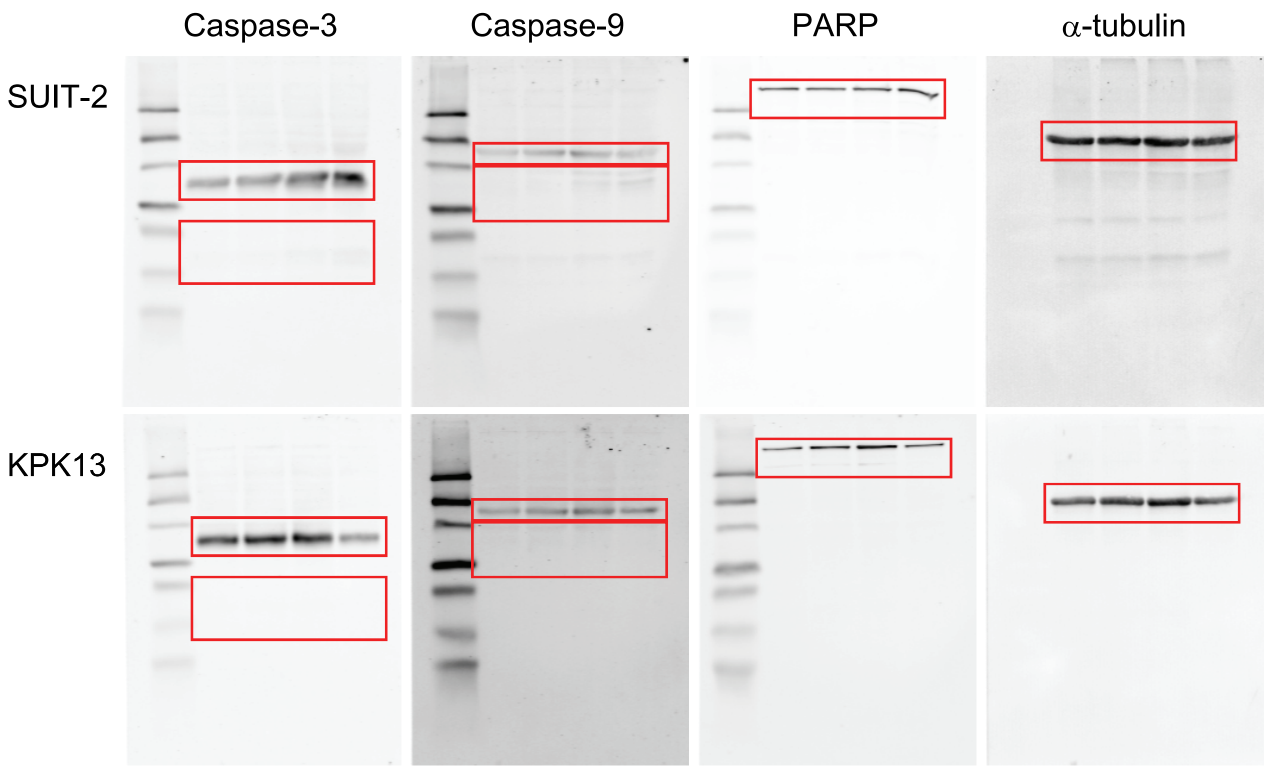

Supplementary Figure S12. Uncropped images of Western blotting if Supplementary Fig. S1

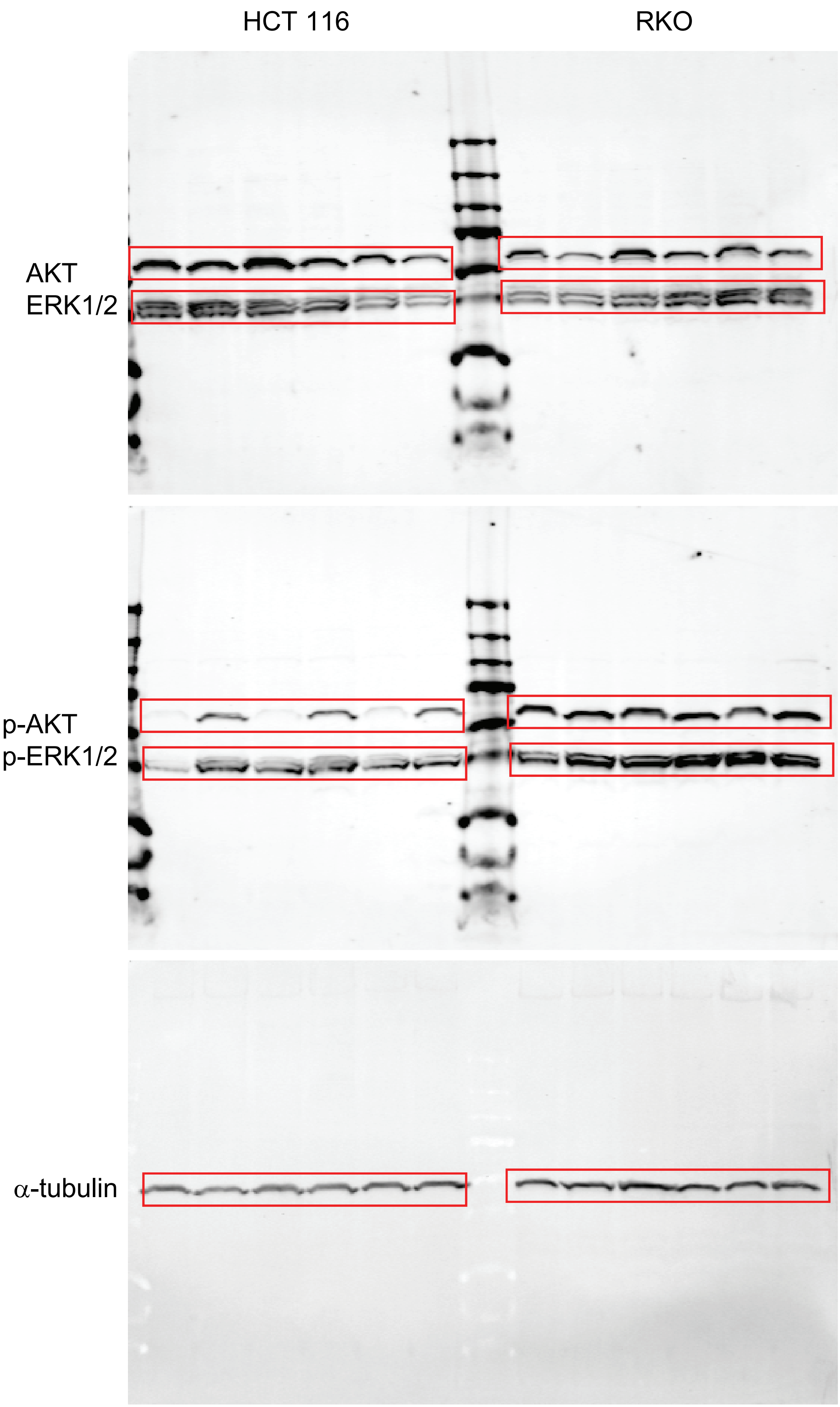

Supplementary Figure S13. Uncropped images of Western blotting of Supplementary Fig. S2b

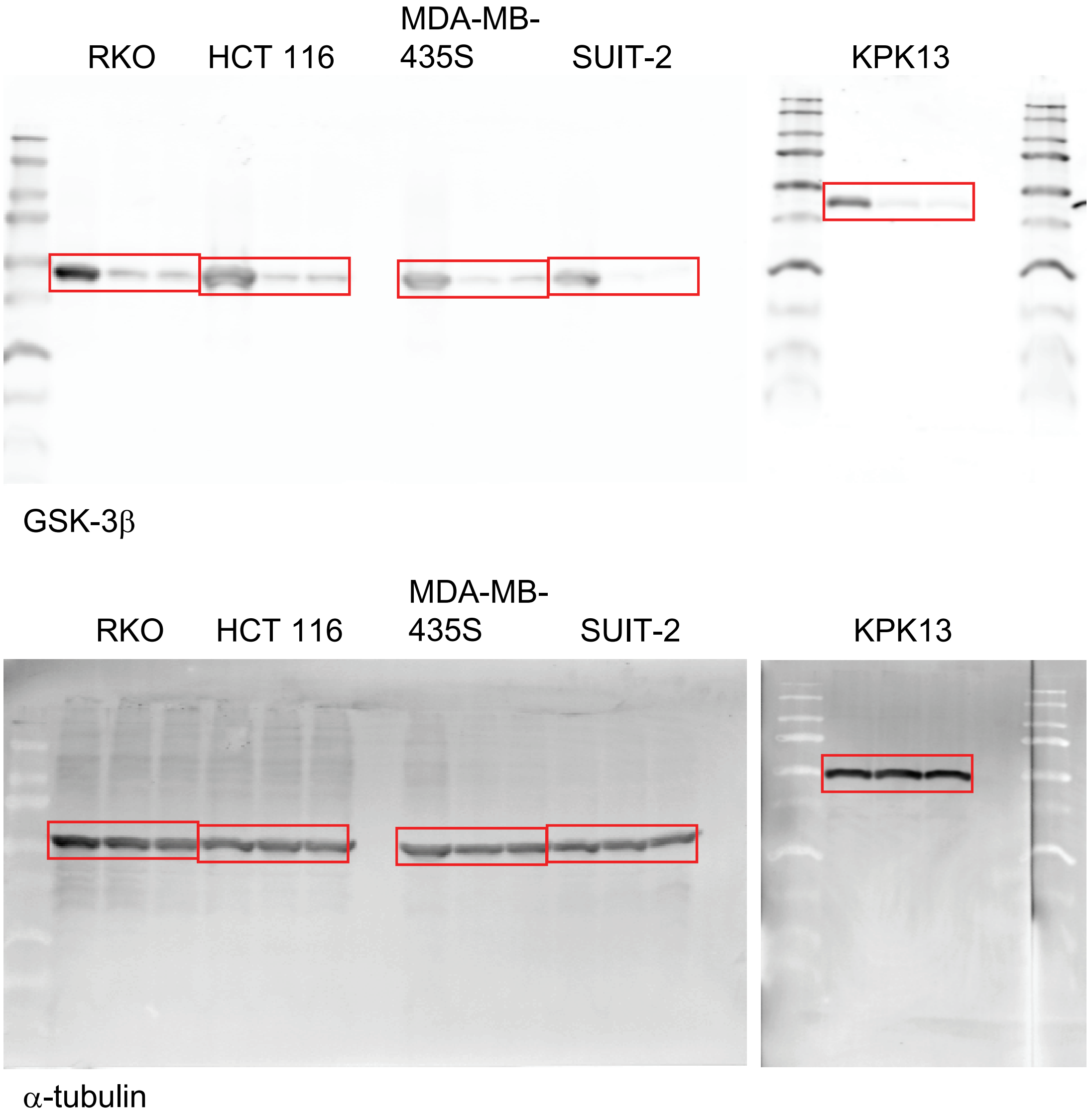

Supplementary Figure S14. Uncropped images of Western blotting of Supplementary Fig. S6a

Antibody name	Supplier	Catalog number	Experiment	Dilution
anti-AKT1	Cell Signaling Technology	#9272	WB	1:1000
anti-pAKT1	Cell Signaling Technology	#4060	WB	1:1000
anti-ERK1/2	Cell Signaling Technology	#9102	WB	1:1000
anti-pERK1/2	Cell Signaling Technology	#3510	WB	1:1000
anti-pSTAT3	Cell Signaling Technology	#9131	WB	1:1000
anti-caspase3	Cell Signaling Technology	#9662	WB	1:1000
anti-caspase9	Cell Signaling Technology	#9502	WB	1:1000
anti-PARP	Cell Signaling Technology	#9532	WB	1:1000
anti-α-tubulin	Sigma	A2066	WB	1:1000
anti-p53	Santa Cruz	sc-6243	WB	1:100
anti-β-catenin	BD	610153	WB	1:1500
anti-mouse IgG, IRDye 800CW	LI-COR Biosciences	926-32210	WB	1:5000
anti-rabbit IgG, IRDye 680RD	LI-COR Biosciences	926-68071	WB	1:5000
anti-α-tubulin	Sigma	A2066	IC	1:100
anti-γ-tubulin	Sigma	T6557	IC	1:500
anti-GSK3β	Cell Signaling Technology	#12456	IC	1:200
anti-pGSK3β	Cell Signaling Technology	#5558	IC	1:400
anti-mouse IgG, AlexaFluor 488	Life Technologies	A-11029	IC	1:400
anti-rabbit IgG, DyLight 649	Rockland Immunochemicals	610-143-121	IC	1:100

Supplementary TableS1. List of antibodies used.
WB; western blot, IC; immunocytofluorescent staining.
